# Assessing the self-reported honesty threshold in adolescent epidemiological research: comparing supervised machine learning and inferential statistical techniques

**DOI:** 10.1186/s12874-023-02035-y

**Published:** 2023-09-21

**Authors:** Janaka V. Kosgolla, Douglas C. Smith, Shahana Begum, Crystal A. Reinhart

**Affiliations:** School of Social Work, University of Illinois Urbana-Champaign, 1010 W. Nevada St, Urbana, IL 61801 USA

**Keywords:** Adolescents, Substance use, Self-reported honesty, Machine learning, Response validity, Epidemiological surveys

## Abstract

**Background:**

Epidemiological surveys offer essential data on adolescent substance use. Nevertheless, the precision of these self-report-based surveys often faces mistrust from researchers and the public. We evaluate the efficacy of a direct method to assess data quality by asking adolescents if they were honest. The main goal of our study was to assess the accuracy of a self-report honesty item and designate an optimal threshold for it, allowing us to better account for its impact on point estimates.

**Methods:**

The participants were from the 2020 Illinois Youth Survey, a self-report school-based survey. We divided the primary dataset into subsets based on responses to an honesty item. Then, for each dataset, we examined two distinct data analysis methodologies: supervised machine learning, using the random forest algorithm, and a conventional inferential statistical method, logistic regression. We evaluated item thresholds from both analyses, investigating probable relationships with reported fake drug use, social desirability biases, and missingness in the datasets.

**Results:**

The study results corroborate the appropriateness and reliability of the honesty item and its corresponding threshold. These contain the agreeing honesty thresholds determined in both data analyses, the identified association between reported fake drug use and lower honesty scores, increased missingness and lower honesty, and the determined link between the social desirability bias and honesty threshold.

**Conclusions:**

Confirming the honesty threshold via missing data analysis also strengthens these collective findings, emphasizing our methodology’s and findings’ robustness. Researchers are encouraged to use self-report honesty items in epidemiological research. This will permit the modeling of accurate point estimates by addressing questionable reporting.

**Supplementary Information:**

The online version contains supplementary material available at 10.1186/s12874-023-02035-y.

## Background

Epidemiological surveys provide much-needed data to public health officials on substance use. Data can be used to determine if policy changes, such as allowing medical or recreational cannabis, increase adolescent use [1, 2]. Additionally, epidemiological studies monitor emerging and trendy substances (i.e., synthetic marijuana, kratom) that, while rare, may result in drastic consequences [3, 4]. Monitoring the use of substances helps evaluate the effectiveness and swiftness of public health responses. Finally, epidemiological studies provide longitudinal trend data, allowing a long-term view of the ebbs and flows of youth substance use.

Despite the critical need for epidemiological surveys, their veracity is continually challenged due to reliance on self-report. Self-reported data are sometimes distrusted by the school principals who allow its collection [5]. Without confidence in such data, public health officials and the lay public may not make informed substance use prevention and treatment decisions. Worse yet, the stakes could involve the erosion of trust between researchers and the public, which has trended downward recently [6].

Scalable methods could improve the validity of self-reports in extensive epidemiological studies. In clinical research with smaller samples than those from epidemiological surveys, it is possible to have interviewers clarify discrepancies in self-report data [7], combine parental reports with youth self-report [8], or collect biological specimens (i.e., urine, hair, or saliva tests) from corresponding self-report data [9]. However, these strategies are impractical for surveys that collect tens of thousands of anonymous adolescent responses. Furthermore, models that capture indifferent, directional, and extreme responses [10] cannot fully account for the complexity of adolescent substance use behaviors or the potential biases in self-report data. Numerous researchers have long speculated that over-reporting of substance use is a problem in self-report adolescent surveys [11–15] which amplifies the need for research on honesty.

Because of their large size, sensitivity analyses on point estimates from epidemiological surveys may be robust to small amounts of invalid data. However, increased problems may exist in obtaining accurate estimates of the prevalence of rare phenomena or their associations with other variables. For example, after eliminating various percentages of data suspected to be derived from mischievous responders, associations between self-reported gender minority status and various mental health problems were significantly reduced in magnitude [16]. In addition, many phenomena of interest in epidemiological surveys on substance use involve data with low base rates in the population. Some examples include establishing the prevalence and correlates of opioid use during high school, determining the association between gender identity (i.e., transgender vs. cisgender) and substance use, or establishing the number of youth ages 12–18 who consider themselves to be in recovery from substance use [17–19].

Furthermore, at the local level, where prevention planning occurs with a much smaller sample size (e.g., 200 adolescents), invalid responses could result in ineffective program planning. Examples may include directing substance use prevention resources toward overestimated problems. Conversely, prevention planning could fail to recognize and prevent a rare, underestimated problem occurring in hot spots. Increasing confidence in the validity of epidemiological surveys on adolescent substance use benefits researchers and prevention specialists.

A straightforward method to assess validity is asking adolescent survey respondents if they were honest. This low-burden method could identify youth who exhibited various response sets, such as mischievous responses [16], underreporting, or overreporting. Several studies have evaluated the effectiveness of self-reported honesty items and scales using inferential statistical studies [20–26].

However, existing literature does not deliver integrate the use of supervised machine learning with traditional inferential statistical methods. In general, machine learning approaches automate analyses with some degree of training an algorithm. This approach is suitable in situations with large data sets. In supervised machine learning, the output is known (honest vs. dishonest) and random forest learning helps to classify the groups based on the known output [27]. In the present study, we use machine learning to scan the entire dataset for variables with the strongest associations with substance use severity scores.

Traditional inferential statistical methods require researchers to specify important variables a priori and require that basic assumptions are met. These include normality of errors, linearity of variable relationships, absence of multicollinearity, homoscedasticity, and independence of observations. Contrariwise, supervised machine learning does not assume specific data distribution and can manage non-linear relationships between variables without sticking to the assumptions above.

We assume that these two methods may yield inherently different thresholds for our self-report honesty item due to their fundamental differences described above. Thus, should the findings converge across machine learning and inferential statistical approaches, it could increase our confidence in the validity of the honesty item and its designated threshold.

While the convergence of the results of the two analytical methods can increase confidence, further validation is needed to ensure suitability and precision - both methods could be consistently incorrect. Therefore, it is vital to utilize further validation techniques. Hence, we strengthened our results by including analyses on social desirability biases, using a fake drug question and missingness.

## Methods

### Data and participants

This study used the 2020 Illinois Youth Survey (IYS) data, a biennial self-reported survey funded by the Illinois Department of Human Services (IDHS) that collects responses from 8th, 9th, 10th, 11th, and 12th-grade adolescents. The survey includes adolescents’ responses about their consumption of substances, perceptions of substance use, family and school support, and health and nutritional habits. In 2020, 616 Illinois schools registered voluntarily to participate in this survey, and 125,067 adolescents took the survey. The survey was administered in school settings via online or paper/pencil format.

### Measures

#### Honesty

At the end of the survey, adolescents responded to the item “How honest were you in filling out this survey?“ on a Likert scale (1: Very honest, 2: Honest pretty much of the time, 3: Honest some of the time, 4: Honest once in a while, and 5: Not honest at all).

#### CRAFFT scale

The widely used CRAFFT scale (Car, Relax, Alone, Forget, Friends, Trouble) measured substance use problem severity. The CRAFFT is a six-item screening measure (1: yes, 0: no; range = 0–6) with high sensitivity for detecting the presence of substance use disorders or heavy cannabis use at a cutoff of two or higher [28, 29]. Thus, we created a dichotomous variable indicating whether or not (1: yes, 0: no) participants met the recommended CRAFFT cutoff.

Furthermore, other scales, alcohol use, and binge drinking during the past 30 days/past year, were measured on the Likert scale (1= “0 occasions” to 6 = “20 or more occasions”). Age was measured on a continuous scale. All the other items mentioned below (unless noted) are dichotomous indicators: been drunk or high at school, drove a car after using marijuana, got alcohol from a friend, first-time alcohol use within past year, got alcohol from a party, perceived prevalence of alcohol use at school (0-100%), got alcohol from parents w/o their permission, perceived risk of marijuana use, recovery problem solved, drove a car after drinking alcohol, felt bad about gambling, suicidal ideation, experienced depression, been drunk or high at school, sold illegal drugs at school, had a fight post/while drinking alcohol, been hurt/injured post/while drinking alcohol, and victim of a violent crime post/while drinking alcohol. We performed a supervised machine-learning analysis employing all observed parameters from the IYS survey. Nevertheless, we only described the measures related to the dependent parameter to retain a concise and direct report. For all measures, item descriptions and response options can be found in supplemental materials (please refer to additional file 1).

### Data and participants

#### Missing data handling

We performed Little’s MCAR test on the primary dataset to determine whether the data were missing at random (MAR), not missing at random (MNAR), or missing completely at random (MCAR). The test showed that the data was indeed MCAR, with a non-significant result (missing patterns = 1710, chi-square = 35,000 df = 342,000, p-value = 1.00). Hence, we used multiple imputation methods to impute missing data. We began with an initial imputation using the mice R statistical software package v3.14.0 [30] to determine the best method based on the parameter measurement scales. Based on predictorMatrix results, predictive mean matching (pmm) was the most appropriate imputation method. Subsequently, we executed the imputation procedure.

#### Subsampling

In IYS 2020, respondents differed across the honesty levels: 72,653 were Very Honest (VH), 26,068 reported being Pretty Honest (PH), 4,916 said they were Sometimes Honest (SH), and 3,522 marked being Rarely Honest (RH). This latter group combined individuals that said they were honest “once in a while” or “not at all.” We assume they are equally invalid and combining them allowed for an adequate analytic sample. We employed a random subsampling strategy to enhance the robustness of our analyses. From these initial groups, VH was subsampled to a size of 5,000; likewise, PH was also decreased to a sample size of 5,000. Due to their smaller counts, the SH and RH datasets stayed unchanged. This approach was used to achieve more balanced sample sizes across the honesty levels, thereby reducing possible biases in our analyses arising from varying group sizes.

#### Supervised machine learning analysis

Our study employed the Random Forest (RF) method to classify the dependent variable responses using given predictors. RF creates multiple decision trees and engages Gini impurity to optimize the data-splitting procedure. Nevertheless, as decision trees are inclined to bias and overfitting, RF improves robustness through feature randomness and bootstrap aggregation. This process involves randomly sampling data, out-of-bag verification, and random choice of predictors for each tree, providing a more reliable and diversified model [31–35].

The initial step of the RF classification technique is bootstrap sampling. One-third of each bootstrap sample (in-bag data) is then separated for testing purposes (out-of-bag (OOB) data), while the remaining data is used for training the model (building the decision trees). The next step comprises feature randomness. As the fourth step, decision trees are built for each training bootstrapped dataset by computing Gini impurity and recursively dividing the training data into subsets where each subset becomes a node in the decision tree. Gini impurity can be computed for items with *J* classes as:

1 $$Gini\left(t\right)=1-\sum\, ^{J}_{i=1}{p(i|t)}^2$$

where:

*Gini(t)*: Gini index for node *t*,

*p(i|t)* is the ratio of the samples belonging to class *i* for node *t*, and *J* is the number of classes. Leaf nodes are determined when only one observation is left in the splitting process.

As an outcome, the RF algorithm calculates the mean decrease in the Gini impurity index, demonstrating input parameter importance concerning the response parameter. Decreased Gini impurity measures the Gini impurity reduction due to including an input parameter to the decision tree. The mean decrease in Gini impurity is the average reduction of Gini impurity for all the decision trees in the RF. A higher mean decrease of Gini impurity for an input parameter indicates that adding that parameter improves the model purity (accuracy), implying that the input parameter is a significant predictor. Therefore, we utilized the mean decrease Gini impurity scores of RF models to evaluate the importance of input parameters. Finally, testing is performed for each decision tree with the OOB data, and the misclassification rate (OOB error rate) is computed.

In our RF analysis, the CRAFFT scale was the dependent parameter and all other observed parameters in the survey pertaining to alcohol and marijuana use were independent predictors. This analysis was completed using R open-source packages randomForest v4.7-1.1, reprtree, caret, and rfPermute v2.5.1 [36–39]. Initially, we conducted a sensitivity analysis for the RF model to determine the best-fitting model parameters. We used the OOB error and the area under the receiver operating characteristic (ROC AUC) to determine the best fit.

#### Inferential statistical analysis

Logistic regression is a statistical method to model dichotomous dependent parameters using odds ratios. The data were analyzed using the R stats package (version 3.6.2). We simulated the impact of risk-taking and sensation-seeking behaviors, depression, suicidal ideation, and age on the CRAFFT measure. We selected relevant input parameters (felt bad about gambling, suicidal ideation, experienced depression, been drunk or high at school, sold illegal drugs at school, had a fight post/while drinking alcohol, been hurt/injured post/while drinking alcohol, victim of a violent crime post/while drinking alcohol, and age), and conducted the generalized linear model analysis for various honesty scales. The model fit was evaluated using the Akaike information criterion (AIC) and Bayesian information criterion (BIC). Additionally, for each parameter in the model, a p-value of less than 0.05 was considered statistically significant.

## Results

The full R code used for the analysis can be located in the supplementary material (please refer to Additional file 2).

### Participant characteristics

The demographics and descriptive statistics for the VH, PH, SH, and RH datasets subsampled within the 2020 IYS dataset are recapitulated in Tables 1 and 2, respectively.

**Table 1:** Demographics

Parameter	Category	Dataset VH (%)	Dataset PH (%)	Dataset SH (%)	Dataset RH (%)
Age	13-year-old	0.2	0.2	0.3	1.3
	14-year-old	5.0	5.0	3.7	3.5
	15-year-old	18.4	19.1	18.7	17.4
	16-year-old	19.0	18.9	19.8	20.1
	17-year-old	31.7	32.1	30.8	28.9
	18-year-old	25.3	24.4	26.1	26.4
	19-year-old	0.4	0.3	0.6	2.4
Gender	Female	49.8	50.6	47.9	32.9
	Male	48.2	47.1	48.8	57.8
	Transgender	0.8	0.9	1.2	3.9
	Do not identify	1.1	1.5	2.0	5.4
Race	White	59.6	52.8	44.7	43.4
	Black	6.8	6.2	8.7	11.5
	Latino	14.1	19.7	25.6	22.0
	Asian American	5.8	6.9	4.2	4.1
	Native American	0.5	0.5	0.6	1.2
	Multi-racial	10.5	11.1	13.3	12.8
	Other	2.7	2.8	2.8	5.0

**Table 2:** Descriptive statistics

Parameter	Dataset VH (%)	Dataset PH (%)	Dataset SH (%)	Dataset RH (%)
Missingness	15.9	15.8	16.7	18
CRAFFT	18.6	21.1	25.4	30.9
Fake drug use	1	1.3	4	15.9
Sold illegal drugs at school	3.3	3.5	6.4	16.2
Been drunk or high at school	7.2	8.7	13.5	21
Drove a car after using marijuana	5.5	5.9	8.1	15.4
Victim of a violent crime (PD)	1	1.2	3.1	10.9
Had a fight (PD)	4	4.7	6.5	13.6
Been hurt/injured (PD)	3.1	3.6	6	13
Got alcohol from a friend	17.2	19.8	20.6	22.2
Got alcohol from a party	8.7	9	10.3	13.8
Got alcohol from parents w/o their permission	11.7	14.2	14.8	19.4
Suicidal ideation	46.4	47.1	46.9	45.7
Felt bad about gambling	40.6	41.8	41.4	42.6
Perceived prevalence of alcohol use at school (> 90%)	5.6	5.4	8.9	17.2
Perceived risk of marijuana use	84.1	82.7	73.3	64.5
Resolved a substance use problem	3.8	4.3	7.2	15.1
Alcohol use over past year	33.1	42.8	41.6	35.3
Alcohol use during past 30 days	21.2	28.4	35.4	38.3
Binge drinking during past 30 days	8.3	10.4	17.6	25.4
First time alcohol use within past year	18.2	23.6	25.3	23.5

### RF analysis

We systematically increased the fundamental model parameter values during the sensitivity analysis and reran the rf model to evaluate its performance. According to this iterative process, the optimum number of trees to grow in the model was 2000, and the optimum number of parameters randomly selected as candidates at each split when creating the individual trees was 20. Then, we conducted the analysis for each dataset and reported the OOB and ROC. We assumed that the lower the OOB error, the higher the classification accuracy of the RF model. Furthermore, an area under the curve (AUC) in receiver operator curves (ROC) above 0.9 indicates an outstanding RF model that performs better than random chance, and a value of 1 indicates a perfect model. Thus, we found that all our RF models performed outstandingly (please refer to Table 3).

**Table 3:** OOB and ROC values of RF analysis

Output	Dataset VH	Dataset PH	Dataset SH	Dataset RH
OOB (%)	8.14	10.62	13.02	13.79
AUC	1.00	0.93	0.93	0.93

**Fig. 1:**
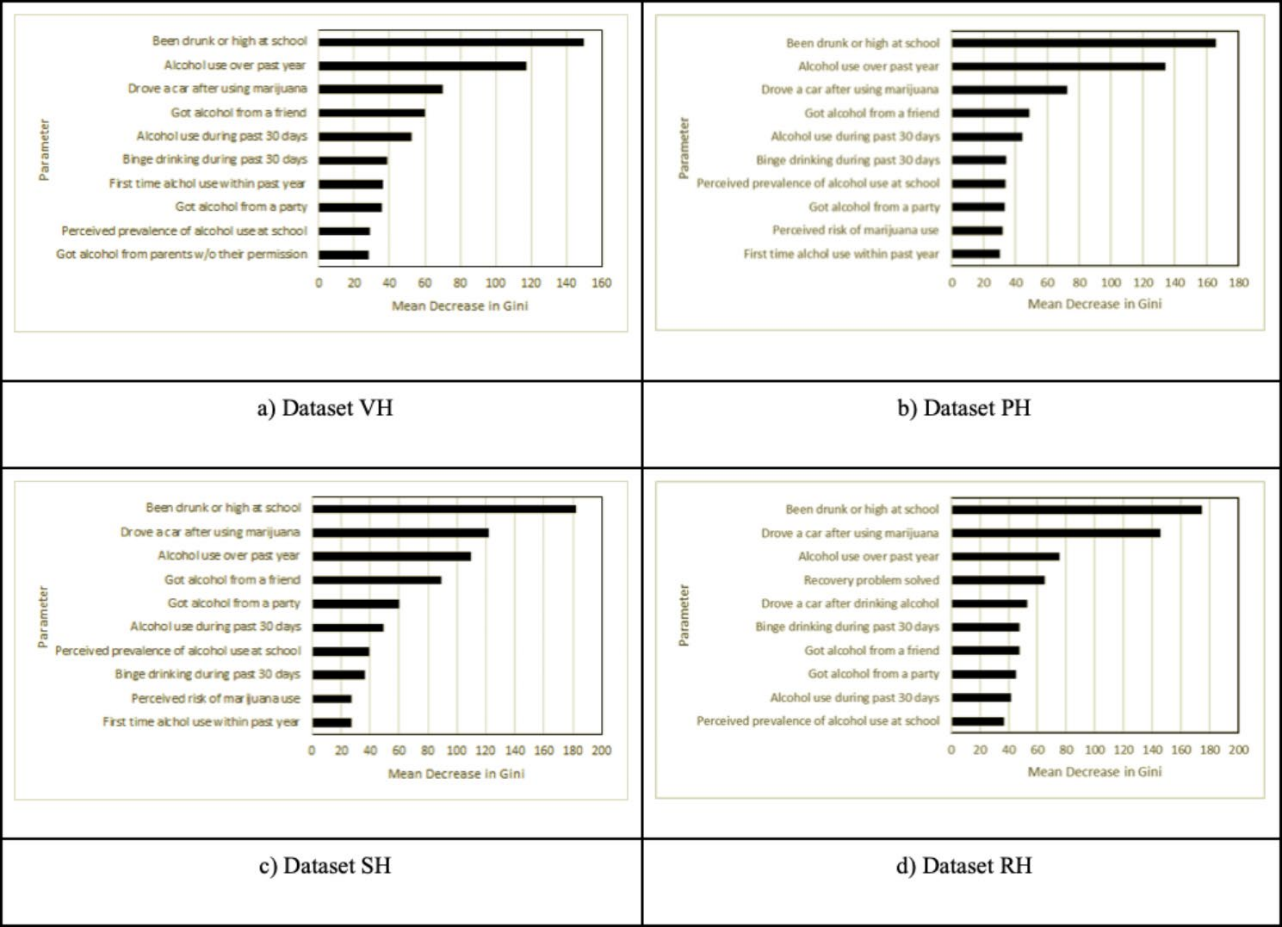
Gini impurities of the random forest model: Analyzing the effects of varying honesty values on the dependent measure, the CRAFFT scale

Additionally, we evaluated the mean decrease Gini impurity scores for all the analysis results to determine the parameter importance. Figure 1 depicts the descending order of significance for input parameters for each honesty level, as computed by the RF model for the CRAFFT scale. This importance is evaluated based on mean decrease in Gini impurity scores attained through the RF analysis. Consequently, the model accurately identified the top six parameters with the highest mean decrease Gini impurity score for datasets VH and PH. However, the model did not accurately evaluate important input parameters for datasets SH and RH.

### Inferential statistical analysis

**Fig. 2:**
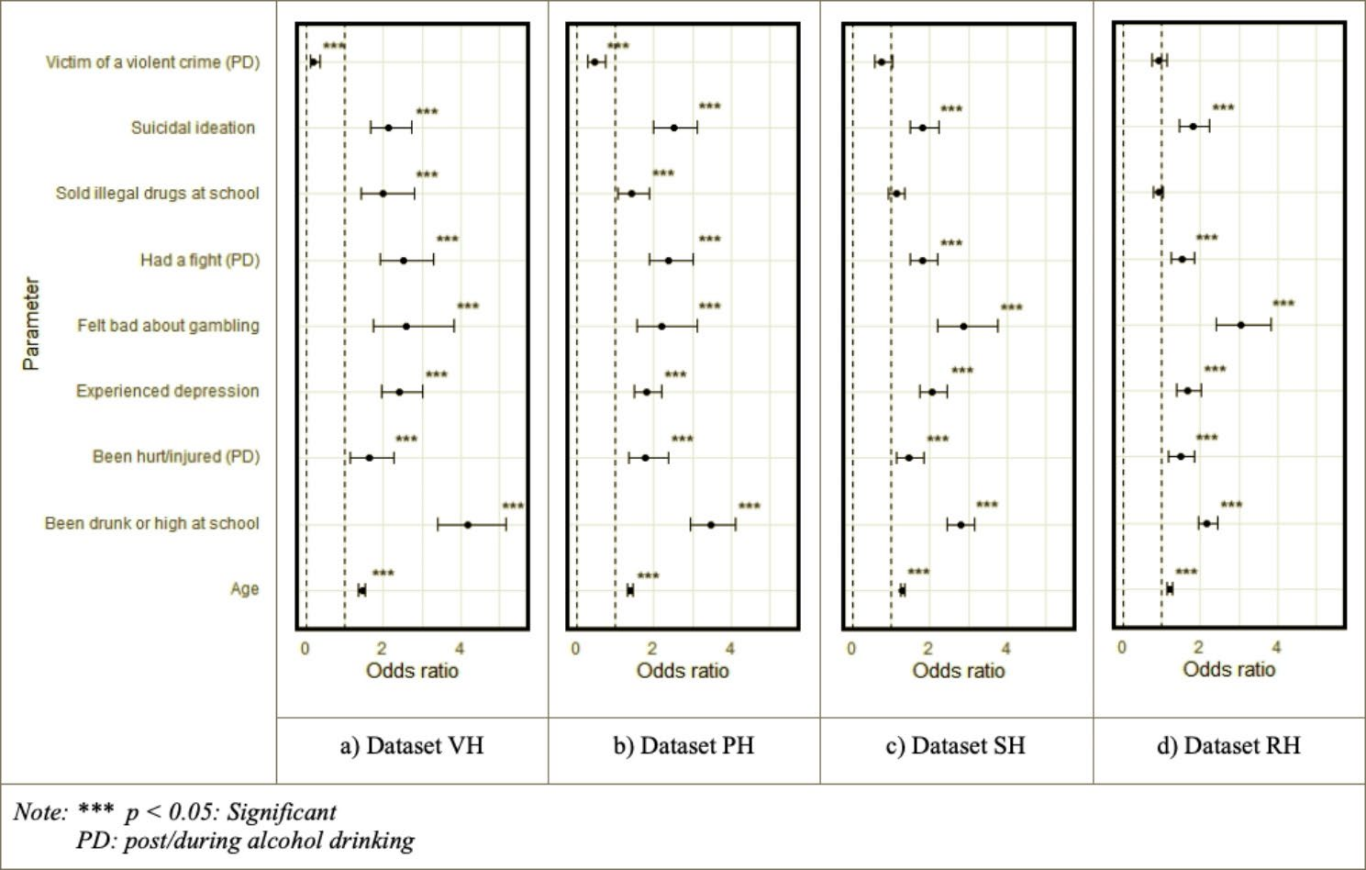
Odds ratios of the generalized linear model: Analyzing the effects of varying honesty values on the dependent measure, the CRAFFT scale

Figure 2 depicts the evaluation of the generalized linear model (logistic regression) that analyzes the effects of different honesty values on the dependent measure, the CRAFFT scale. The fit indices AIC and BIC were: dataset VH: AIC = 2858.25, BIC = -39662.53; dataset PH: AIC = 3427.20, BIC = -39093.58; dataset SH: AIC = 3901.62, BIC = -38619.17; dataset RH: AIC = 3509.98, BIC = -32186.43. Subsequently, we can deduce that inferences made from the analysis became insignificant for parameters “sold illegal drugs at school” and “was a victim of a violent crime post/while drinking alcohol” when the honesty level was below PH.

### Reported use of a fake drug

Also, we evaluated whether honesty level was associated with a higher percentage of youth reporting they used a fake drug, which is included as a validity check for carelessness and mischievous responses. A higher percentage of adolescents reported using a fake drug as youth reported being less honest (please refer to Table 2).

### Missingness and honesty

**Fig. 3:**
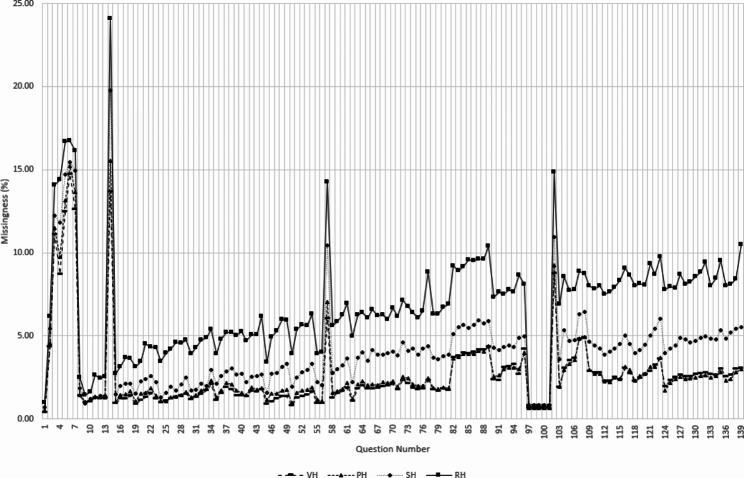
Missing data percentage for each survey question for different honesty levels

We studied missing data patterns for all the parameters for different honesty levels. As illustrated in Fig. 3 (refer to the additional file 1 for a comprehensive view of the questionnaire items), SH and RH responses demonstrated a substantial increase in the percentages of missing data as the survey progressed compared to their VH and PH counterparts. Similarly, the overall missing data percentage was higher for SH and RH responses, revealing a negative association between data missingness and self-reported honesty.

## Discussion

In the RF model used in the SH and RH datasets, the responses from adolescents with lower honesty levels detrimentally influenced the model’s accuracy and validity. To elaborate, the RF model failed to approximate the crucial input parameters in these datasets accurately. This finding is echoed by the inferential statistical model, underpinning its validity.

Additionally, the inferential statistics model demonstrated inconsistencies in responses from adolescents classified as SH and RH, especially for the parameters “sold illegal drugs at school” and “was the victim of violent crime post/while or after drinking alcohol.” These parameters are deemed the most stigmatizing among the independent parameters in the model, which can be related to the impact of social desirability bias on the honesty threshold. This relationship also aligns with the threshold demarcated from both analytical models, emphasizing the association between the honesty threshold and reaction to socially unpleasant scenarios [40, 41].

According to the missing data analysis, less honest respondents were also likelier to leave incomplete responses. This association and the missingness threshold observed beginning at the SH level further validate our findings from two separate analyses.

The consistency of honesty thresholds emanated from both analyses, the relationship of reported fake drug use with lower honesty scores, the determined association between honesty threshold and social desirability bias, and finally, the confirmation of the honesty threshold through missing data analysis all collectively emphasize the suitability and reliability of the honesty scale and its relevant threshold. The results suggest that carefully applying the honesty scale’s threshold in the primary dataset requires the deletion of SH and RH responses. However, conceptually, it may be difficult for researchers to imagine that youth who provide questionable responses to an epidemiological survey would recant at the end when presented with a self-reported honesty item. Nevertheless, these analyses support the occurrence of this phenomenon. These findings also indicate that it is critical to consider case selection rules and data quality when using epidemiological data for studies on rarely used substances such as heroin.

### Sensitivity testing

As only 8% of the responders in the IYS dataset were recognized as sometimes honest and rarely honest, their removal may have a minimal impact on aggregated point estimates. However, deletion may be more challenging to address when dealing with smaller datasets where less honest responses constitute a more significant proportion. This is also the scenario when investigators subset data from extensive epidemiological studies to investigate rare phenomena among youth (e.g., heroin use, recovery, being transgender). For example, if, as we saw here, there was more overreporting on a rare phenomenon, it would create difficult decisions for researchers using a small subset. They would need to choose between potentially lowering the analytic sample size of some groups of interest (e.g., transgender youth). This may improve data quality but lower statistical power for analyses. In such a case, it is advisable to utilize sensitivity analysis (see [16] for more details) to designate an appropriate threshold for excluding invalid responses, thereby confirming the validity and integrity of the findings.

Furthermore, when using honesty item, the direction of biases can become observable, as we have demonstrated in our RF (Fig. 1) and logistic regression analyses (Fig. 2) and descriptive (Table 2). So, this can inform what strategies are needed to address biases introduced by dishonest reporting. More guidance, however, is needed on what percent of data can be eliminated, so we encourage future simulation studies on this topic. We also encourage researchers to investigate other ways to model point estimates that do not rely on deleting data from respondents reporting low honesty. To our knowledge, there are no studies that have empirically validated such corrective procedures in adolescent epidemiological research. This is especially important in smaller subsets of data drawn from large epidemiological surveys.

### Limitations

Our study is limited in the following ways. First, the 2020 IYS did not represent all Illinois youth, as it ended prematurely during the COVID-19 pandemic. Second, no biological measures were available to integrate into our models, which may have provided additional measures for testing the construct validity of the honesty item (although we note that biological testing in an epidemiological survey may be cost-prohibitive). Third, our honesty measure was a single item that asked youth about their honesty across the entire survey. Some researchers are reluctant to use a single item, preferring scales that can provide more breadth of measurement. However, we note that the single item used here was extensively validated in this study as an indicator of data quality.

Furthermore, there are examples of single items with better predictive validity than entire scales [42, 43]. The intrinsic subjectivity, concealed aspects, and variability of social desirability bias pose a substantial challenge in its measurement and identification. Also, there are examples of single items with better predictive validity than entire scales [42, 43]. The intrinsic subjectivity, concealed aspects, and variability of social desirability bias pose a substantial challenge in its measurement and identification. Also, our survey responses are provided anonymously. Social desirability bias is inversely correlated to anonymity in self-reported survey responses. Nevertheless, it is essential to recognize that anonymity is not a comprehensive solution and cannot eliminate all self-reporting biases [44]. Consequently, we cannot confirm the association between the social desirability bias and the honesty threshold.

Finally, evaluating honesty in adolescent self-reported surveys is difficult due to subjectivity. Their cognitive development stage can obscure the sense of abstract concepts such as honesty. Even with anonymity, there could be concerns of repercussions directing to less honest responses. Smaller datasets further complicate the problem as generalizing results is challenging. Despite such issues, numerous measures and indicators can help evaluate the reliability and validity of the honesty scale for smaller datasets.

## Conclusions

Despite the limitations, our study presents a substantial contribution to the field. It expands the confirmation of this honesty item’s reliability, relevance, and associated threshold by involving two analytical methods. Moreover, the designation of a recommended honesty threshold was further substantiated by equipping fake drug use, social desirability bias, and missingness in data. Hence, our research delivers a more robust test of the honesty item’s performance.

In conclusion, we encourage researchers using epidemiological data to consider the effects of dishonesty as a potential limitation. A self-reported honesty item may be a scalable solution to improving data quality in large epidemiological surveys without increasing respondent burden. Researchers are encouraged to set a suitable threshold for their specific population. Identifying and modeling the impact of invalid responding is advised for large epidemiological studies, as they influence public health decisions.

### Electronic supplementary material

Below is the link to the electronic supplementary material.

**Additional file 1**. 2020 IYS questions and response choices. For all measures considered in the current study, question text and response choices in the IYS survey are included in this file. This file can be used to identify parameter names and corresponding survey question numbers.

**Additional file 2**. R code for the analysis. The complete R code that was used for the analysis is included in this file.

## Data Availability

Raw data used in this study are available upon request.

## List of abbreviations

AIC: Akaike information criterion
BIC: Bayesian information criterion
CRAFFT: Car, Relax, Alone, Forget, Friends, Trouble
IDHS: Illinois Department of Human Services
IYS: Illinois Youth Survey
MAR: Missing at random
MCAR: Missing completely at random
MNAR: Missing not at random
OOB: Out-of-bag
PH: Pretty honest
RF: Random forest
RH: Rarely honest
ROC AUC: Receiver operating characteristic area under the curve
SH: Sometimes honest
SUPR: Substance Use Prevention and Recovery
VH: Very honest
