# Supplementary material for: Assessing the self-reported honesty threshold in adolescent epidemiological research: comparing supervised machine learning and inferential statistical techniques
Source: BMC Med Res Methodol. 2023 Sep 21;23:210. doi: 10.1186/s12874-023-02035-y (PMC10512612; doi:10.1186/s12874-023-02035-y)
Supplement: Supplementary file 1 — Additional file 1. 2020 IYS questions and response choices. For all measures considered in the current study, question text and response choices in the IYS survey are included in this file. This file can be used to identify parameter names and corresponding survey question numbers. [file 12874_2023_2035_MOESM1_ESM.pdf]

Title: 2020 IYS questions and response choices

Legend: For all measures considered in the current study, question text and response choices in the IYS survey are included below.

Questions Number	Category/Questions	Question Text	Response Options	Parameter name in the dataset
**Absenteeism**				
1	Absenteeism	About how many days are you absent from school during an entire year?	0 - 9 days<br>10 - 19 days<br>20 - 30 days<br>More than 30 days	absent
**Participation in School Activities**				
2	Participation in School Activities	In which of the following activities do you participate? School sports team	Yes<br>No	act_sspo
3	Participation in School Activities	In which of the following activities do you participate? Other sports	Yes<br>No	act_ospo
4	Participation in School Activities	In which of the following activities do you participate? School clubs	Yes<br>No	act_sclb
5	Participation in School Activities	In which of the following activities do you participate? Service clubs or volunteer projects (e.g., Scouting, 4H)	Yes<br>No	act_svol
6	Participation in School Activities	In which of the following activities do you participate? Other activity clubs (e.g., Boys & Girls, YMCA, etc.)	Yes<br>No	act_oacl
7	Participation in School Activities	In which of the following activities do you participate? Church or other faith-based youth group	Yes<br>No	act_ygrp
**Employment**				
8	Employment	On the average over the school year, how many hours per week do you work in a paid or unpaid job?	None<br>5 or less hours<br>6 to 10 hours<br>11 to 15 hours<br>16 to 20 hours<br>21 to 25 hours<br>26 to 30 hours<br>More than 30 hours	wk_pay
**Ease of Access**				
9	Ease of Access	If you wanted to get beer, wine, or hard liquor (e.g., vodka, whiskey, or gin) how easy would it be for you to get some?	Very hard<br>Sort of hard<br>Sort of easy<br>Very easy	alco_get
10	Ease of Access	If you wanted to get e-cigarettes or other vaping products, how easy would it be for you to get some?	Very hard<br>Sort of hard<br>Sort of easy<br>Very easy	ecig_get

11	Ease of Access	If you wanted to get cigarettes, how easy would it be for you to get some?	Very hard<br>Sort of hard<br>Sort of easy<br>Very easy	toba_get
12	Ease of Access	If you wanted to get marijuana, how easy would it be for you to get some?	Very hard<br>Sort of hard<br>Sort of easy<br>Very easy	pot_get
13	Ease of Access	If you wanted to get prescription drugs not prescribed to you, how easy would it be for you to get some?	Very hard<br>Sort of hard<br>Sort of easy<br>Very easy	rx_get
14	Ease of Access	If you wanted to get opioid medications from your home, how easy would it be for you to get some? Opioids include methadone, opium, morphine, fentanyl, Vicodin, MS Contin, codeine, Demerol, Roxicodone, hydrocodone (Lortab, Lorcet, Norco), Suboxone, OxyContin, Percocet, Tylox, Percodan, Ultram, and tramadol.	Very hard<br>Sort of hard<br>Sort of easy<br>Very easy	opmeds_get
**Adult Disapproval**				
15	Adult Disapproval	How wrong would most adults (over 21) in your community think it is for kids your age: to use marijuana?	Very wrong<br>Wrong<br>A little bit wrong<br>Not wrong at all	dis_pot
16	Adult Disapproval	How wrong would most adults (over 21) in your community think it is for kids your age: to drink alcohol?	Very wrong<br>Wrong<br>A little bit wrong<br>Not wrong at all	dis_alc
17	Adult Disapproval	How wrong would most adults (over 21) in your community think it is for kids your age: to use e-cigarettes or other vaping products?	Very wrong<br>Wrong<br>A little bit wrong<br>Not wrong at all	dis_ecig
18	Adult Disapproval	How wrong would most adults (over 21) in your community think it is for kids your age: to smoke cigarettes?	Very wrong<br>Wrong<br>A little bit wrong<br>Not wrong at all	dis_tob
**Personal Disapproval**				
19	Personal Disapproval	How wrong do you think it is for someone your age to: drink beer, wine, or hard liquor (e.g., vodka, whiskey or gin) regularly?	Very wrong<br>Wrong<br>A little bit wrong<br>Not wrong at all	wrng_alc
20	Personal Disapproval	How wrong do you think it is for someone your age to: use e-cigarettes or other vaping products?	Very wrong<br>Wrong<br>A little bit wrong<br>Not wrong at all	wrng_ecig

21	Personal Disapproval	How wrong do you think it is for someone your age to: smoke cigarettes?	Very wrong<br>Wrong<br>A little bit wrong<br>Not wrong at all	wrng_cig
22	Personal Disapproval	How wrong do you think it is for someone your age to: use marijuana?	Very wrong<br>Wrong<br>A little bit wrong<br>Not wrong at all	wrng_pot
23	Personal Disapproval	How wrong do you think it is for someone your age to: use prescription drugs not prescribed to them?	Very wrong<br>Wrong<br>A little bit wrong<br>Not wrong at all	wrng_rx
**Parent Disapproval**				
24	Parent Disapproval	How wrong do your parents feel it would be for you to: drink beer, wine, or hard liquor (e.g., vodka, whiskey, or gin) regularly (at least once or twice a month)?	Very wrong<br>Wrong<br>A little bit wrong<br>Not wrong at all	parw_alc
25	Parent Disapproval	How wrong do your parents feel it would be for you to: have one or two drinks of an alcoholic beverage nearly every day?	Very wrong<br>Wrong<br>A little bit wrong<br>Not wrong at all	dparw_alc
26	Parent Disapproval	How wrong do your parents feel it would be for you to: use e-cigarettes or other vaping products?	Very wrong<br>Wrong<br>A little bit wrong<br>Not wrong at all	parw_ecig
27	Parent Disapproval	How wrong do your parents feel it would be for you to: smoke tobacco?	Very wrong<br>Wrong<br>A little bit wrong<br>Not wrong at all	parw_tob
28	Parent Disapproval	How wrong do your parents feel it would be for you to: use marijuana?	Very wrong<br>Wrong<br>A little bit wrong<br>Not wrong at all	parw_pot
29	Parent Disapproval	How wrong do your parents feel it would be for you to: use prescription drugs not prescribed to you?	Very wrong<br>Wrong<br>A little bit wrong<br>Not wrong at all	dparw_rx
**Peer Disapproval**				
30	Peer Disapproval	How wrong do your friends feel it would be for you to: have one or two drinks of an alcoholic beverage nearly every day?	Very wrong<br>Wrong<br>A little bit wrong<br>Not wrong at all	dfr_alc

31	Peer Disapproval	How wrong do your friends feel it would be for you to: use e-cigarettes or other vaping products?	Very wrong<br>Wrong<br>A little bit wrong<br>Not wrong at all	dfr_ecig
32	Peer Disapproval	How wrong do your friends feel it would be for you to: smoke tobacco?	Very wrong<br>Wrong<br>A little bit wrong<br>Not wrong at all	dfr_tob
33	Peer Disapproval	How wrong do your friends feel it would be for you to: use marijuana?	Very wrong<br>Wrong<br>A little bit wrong<br>Not wrong at all	dfr_pot
34	Peer Disapproval	How wrong do your friends feel it would be for you to: use prescription drugs not prescribed to you?	Very wrong<br>Wrong<br>A little bit wrong<br>Not wrong at all	dfr_rx
**Perceived Risk of Harm**				
35	Perceived Risk of Harm	How much do you think people risk harming themselves (physically or in other ways) if they: smoke one or more packs of cigarettes per day?	No risk<br>Slight risk<br>Moderate risk<br>Great risk	rsk_1pac
36	Perceived Risk of Harm	How much do you think people risk harming themselves (physically or in other ways) if they: use e-cigarettes or other vaping products?	No risk<br>Slight risk<br>Moderate risk<br>Great risk	rsk_ecig
37	Perceived Risk of Harm	How much do you think people risk harming themselves (physically or in other ways) if they: take one or two drinks of an alcoholic beverage (beer, wine, liquor) nearly every day?	No risk<br>Slight risk<br>Moderate risk<br>Great risk	rsk_alc
38	Perceived Risk of Harm	How much do you think people risk harming themselves (physically or in other ways) if they: have five or more drinks of an alcoholic beverage once or twice a week?	No risk<br>Slight risk<br>Moderate risk<br>Great risk	rsk_bng
39	Perceived Risk of Harm	How much do you think people risk harming themselves (physically or in other ways) if they: use marijuana once or twice a week?	No risk<br>Slight risk<br>Moderate risk<br>Great risk	rsk_dlyp
40	Perceived Risk of Harm	How much do you think people risk harming themselves (physically or in other ways) if they: use prescription drugs that are not prescribed to them?	No risk<br>Slight risk<br>Moderate risk<br>Great risk	drsk_rx
**Seen as Cool**				

41	Seen as Cool	What are the chances you would be seen as cool if you: used e-cigarettes or other vaping products?	No or very little chance<br>Little chance<br>Some chance<br>Pretty good chance<br>Very good chance	cool_ecig
42	Seen as Cool	What are the chances you would be seen as cool if you: smoked cigarettes?	No or very little chance<br>Little chance<br>Some chance<br>Pretty good chance<br>Very good chance	cool_cig
43	Seen as Cool	What are the chances you would be seen as cool if you: began drinking alcohol regularly, that is, at least once or twice a month?	No or very little chance<br>Little chance<br>Some chance<br>Pretty good chance<br>Very good chance	cool_alc
44	Seen as Cool	What are the chances you would be seen as cool if you:-used marijuana?	No or very little chance<br>Little chance<br>Some chance<br>Pretty good chance<br>Very good chance	cool_pot
**Depression**				
45	Depression	During the past 12 months did you ever feel so sad or hopeless almost every day for two weeks or more in a row that you stopped doing some usual activities?	Yes<br>No	dep_stop
**When, if ever, did you first use?**				
46	When, if ever, did you first use?	When, if ever, did you FIRST: drink more than a sip or two of beer, wine, or hard liquor (e.g., vodka, whiskey, or gin)?	Never have<br>More than 12 months ago<br>During the past 12 months	fUse_alc
47	When, if ever, did you first use?	When, if ever, did you FIRST: smoke a cigarette, even just a puff?	Never have<br>More than 12 months ago<br>During the past 12 months	fUse_cig
48	When, if ever, did you first use?	When, if ever, did you FIRST: use an e-cigarette or other vaping product?	Never have<br>More than 12 months ago<br>During the past 12 months	fUse_ecig

49	When, if ever, did you first use?	When, if ever, did you FIRST: use marijuana?	Never have<br>More than 12 months ago<br>During the past 12 months	fUse_pot
**Past 30 Day Substance Use Prevalence**				
50	Cigarettes	How frequently have you smoked cigarettes during the past 30 days?	Not at all<br>Less than one cigarette per day<br>1-5 cigarettes per day<br>About one-half pack per day<br>About one pack per day<br>More than 1 pack per day	cig_30
51	Smokeless Tobacco	How frequently have you: used smokeless tobacco such as chewing tobacco, snuff, dip, or snus during the past 30 days?	Never<br>Once or twice<br>Once or twice per week<br>About once a day<br>More than once a day	chew_30
52	Tobacco Products	How frequently have you: smoked tobacco products other than cigarettes such as cigars, cigarillos, or little cigars during the past 30 days?	Never<br>Once or twice<br>Once or twice per week<br>About once a day<br>More than once a day	smtob_30
53	E-Cigarettes/Vaping	How frequently have you: used e-cigarettes or other vaping products during the past 30 days?	Never<br>Once or twice<br>Once or twice per week<br>About once a day<br>More than once a day	ecig_30
54	Hookah	How frequently have you: used a hookah or water pipe during the past 30 days?	Never<br>Once or twice<br>Once or twice per week<br>About once a day<br>More than once a day	hookah_30

55	Alcohol	On how many occasions (if any) have you had beer, wine, or hard liquor during the past 30 days?	0 occasions<br>1 - 2 occasions<br>3 - 5 occasions<br>6 - 9 occasions<br>10 - 19 occasions<br>20 or more occasions	alc_30
56	Binge Drinking	Think back over the last two weeks. How many times have you had five or more alcoholic drinks in a row?	None<br>Once<br>Twice<br>3-5 times<br>6-9 times<br>10 or more times	binge
57	Marijuana	On how many occasions (if any) have you used marijuana during the past 30 days?	0 occasions<br>1 - 2 occasions<br>3 - 5 occasions<br>6 - 9 occasions<br>10 - 19 occasions<br>20 or more occasions	pot_30
58	Marijuana	In the past 30 days, have you used marijuana in any of the following ways? Smoked it (in a joint, bong, pipe, blunt)	Yes<br>No	pothow_smk
59	Marijuana	In the past 30 days, have you used marijuana in any of the following ways? Vaporized it (e.g., vapor pen)	Yes<br>No	pothow_vap
60	Marijuana	In the past 30 days, have you used marijuana in any of the following ways? Ate it (in brownies, cakes, candy, etc.)	Yes<br>No	pothow_ate
61	Marijuana	In the past 30 days, have you used marijuana in any of the following ways? Dabbed it	Yes<br>No	pothow_dab
62	Prescription Drugs	During the past 30 days have you used prescription drugs not prescribed to you?	Yes<br>No	drx_30
**Past Year Substance Use Prevalence**				
63	Cigarettes	How frequently have you smoked cigarettes during the past year?	Not at all<br>Less than one cigarette per day<br>1-5 cigarettes per day<br>About one-half pack per day<br>About one pack per day<br>More than 1 pack per day	alc_yr

64	Alcohol	In the past year, on how many occasions (if any) have you: had beer, wine, or liquor?	0 occasions<br>1 - 2 occasions<br>3 - 5 occasions<br>6 - 9 occasions<br>10 - 19 occasions<br>20 or more occasions	inh_yr
65	Inhalants	In the past year, on how many occasions (if any) have you: sniffed glue, breathed the contents of an aerosol spray can, or inhaled other gases or sprays in order to get high?	0 occasions<br>1 - 2 occasions<br>3 - 5 occasions<br>6 - 9 occasions<br>10 - 19 occasions<br>20 or more occasions	anytob_yr
66	Tobacco Products	In the past year, on how many occasions (if any) have you: used any tobacco product including smokeless tobacco, tobacco smoked through cigarettes or cigars/cigarillos, or tobacco used in a hookah water pipe?	0 occasions<br>1 - 2 occasions<br>3 - 5 occasions<br>6 - 9 occasions<br>10 - 19 occasions<br>20 or more occasions	pot_yr
67	Marijuana	In the past year, on how many occasions (if any) have you: used marijuana?	0 occasions<br>1 - 2 occasions<br>3 - 5 occasions<br>6 - 9 occasions<br>10 - 19 occasions<br>20 or more occasions	mdm_yr
68	MDMA/Ecstasy	In the past year, on how many occasions (if any) have you: used MDMA ("ecstasy")?	0 occasions<br>1 - 2 occasions<br>3 - 5 occasions<br>6 - 9 occasions<br>10 - 19 occasions<br>20 or more occasions	lsd_yr
69	LSD	In the past year, on how many occasions (if any) have you: used LSD or other psychedelics?	0 occasions<br>1 - 2 occasions<br>3 - 5 occasions<br>6 - 9 occasions<br>10 - 19 occasions<br>20 or more occasions	coc_yr

70	Cocaine/Crack	In the past year, on how many occasions (if any) have you: used cocaine or crack?	0 occasions<br>1 - 2 occasions<br>3 - 5 occasions<br>6 - 9 occasions<br>10 - 19 occasions<br>20 or more occasions	naz_yr
71	Methamphetamines	In the past year, on how many occasions (if any) have you: used meth (methamphetamine)?	0 occasions<br>1 - 2 occasions<br>3 - 5 occasions<br>6 - 9 occasions<br>10 - 19 occasions<br>20 or more occasions	mth_yr
72	Heroin	In the past year, on how many occasions (if any) have you: used heroin?	0 occasions<br>1 - 2 occasions<br>3 - 5 occasions<br>6 - 9 occasions<br>10 - 19 occasions<br>20 or more occasions	her_yr
73	E-Cigarettes/Vaping	In the past year, on how many occasions (if any) have you: used e-cigarettes or other vaping products?	0 occasions<br>1 - 2 occasions<br>3 - 5 occasions<br>6 - 9 occasions<br>10 - 19 occasions<br>20 or more occasions	ecig_yr
74	Marijuana	In the past year, on how many occasions (if any) have you: used synthetic marijuana (K2, spice, or fake weed)?	0 occasions<br>1 - 2 occasions<br>3 - 5 occasions<br>6 - 9 occasions<br>10 - 19 occasions<br>20 or more occasions	sythpot_yr
75	Poly Drug Use	In the past year, on how many occasions (if any) have you: used marijuana and alcohol at the same time?	0 occasions<br>1 - 2 occasions<br>3 - 5 occasions<br>6 - 9 occasions<br>10 - 19 occasions<br>20 or more occasions	alcpot_yr

76	Alcohol	In the past year, on how many occasions (if any) have you: used alcohol and energy drinks at the same time?	0 occasions<br>1 - 2 occasions<br>3 - 5 occasions<br>6 - 9 occasions<br>10 - 19 occasions<br>20 or more occasions	alcnrgy_yr
77	Prescription Drugs	During the past year have you used prescription drugs NOT PRESCRIBED TO YOU?	Yes<br>No	drx_yr
78	Prescription Drugs	During the past 12 months, how often have you used: prescription pain medicine without a doctor's prescription or differently than how a doctor told you to use it? (Count drugs such as codeine/"lean", Vicodin, OxyContin, hydrocodone, and Percocet.)	Never<br>1-2 times<br>3-5 times<br>6 or more times	ppnorx_yr
79	OTC	During the past 12 months, how often have you used: something you bought in a store to get high? (e.g., cough syrup, etc.)	Never<br>1-2 times<br>3-5 times<br>6 or more times	ocd_yr
80	Prescription Drugs	During the past 12 months, how often have you used: prescription painkillers to get high? (e.g., OxyContin, Vicodin, Lortab, etc.)	Never<br>1-2 times<br>3-5 times<br>6 or more times	pp_yr
81	Prescription Drugs	During the past 12 months, how often have you used: other prescription drugs to get high? (e.g., Ritalin, Adderall, Xanax, etc.)	Never<br>1-2 times<br>3-5 times<br>6 or more times	opd_yr
**While or After Drinking**				
82	While or After Drinking	During the past 12 months, how often have you experienced the following WHILE or AFTER DRINKING ALCOHOL: Performed poorly on a test or important project	Never<br>1-2 times<br>3-5 times<br>6 or more times	wad_test
83	While or After Drinking	During the past 12 months, how often have you experienced the following WHILE or AFTER DRINKING ALCOHOL: Been in trouble with the police	Never<br>1-2 times<br>3-5 times<br>6 or more times	wad_cops
84	While or After Drinking	During the past 12 months, how often have you experienced the following WHILE or AFTER DRINKING ALCOHOL: Damaged property	Never<br>1-2 times<br>3-5 times<br>6 or more times	wad_prop
85	While or After Drinking	During the past 12 months, how often have you experienced the following WHILE or AFTER DRINKING ALCOHOL: Got into an argument or fight	Never<br>1-2 times<br>3-5 times<br>6 or more times	wad_fght

86	While or After Drinking	During the past 12 months, how often have you experienced the following WHILE or AFTER DRINKING ALCOHOL: Been hurt or injured	Never<br>1-2 times<br>3-5 times<br>6 or more times	wad_hurt
87	While or After Drinking	During the past 12 months, how often have you experienced the following WHILE or AFTER DRINKING ALCOHOL: Been a victim of a violent crime	Never<br>1-2 times<br>3-5 times<br>6 or more times	wad_vic
88	While or After Drinking	During the past 12 months, how often have you experienced the following WHILE or AFTER DRINKING ALCOHOL: Been treated in a hospital Emergency Department	Never<br>1-2 times<br>3-5 times<br>6 or more times	wad_ed
89	While or After Drinking	During the past 12 months, how often have you experienced the following WHILE or AFTER DRINKING ALCOHOL: A friend who is about your age said they were worried about your alcohol use	Never<br>1-2 times<br>3-5 times<br>6 or more times	wad_frnd
**CRAFFT Scale**				
90	CRAFFT Scale	During the past 12 months, did you ever use alcohol or drugs to RELAX, feel better about yourself, or fit in?	Yes<br>No	dd_relax
91	CRAFFT Scale	During the past 12 months, did you ever use alcohol or drugs while you were by yourself, ALONE?	Yes<br>No	dd_alone
92	CRAFFT Scale	During the past 12 months, did you ever FORGET things you did while using alcohol or drugs?	Yes<br>No	dd_4get
93	CRAFFT Scale	During the past 12 months, did your family or FRIENDS ever tell you that you should cut down on your drinking or drug use?	Yes<br>No	dd_frnd
94	CRAFFT Scale	During the past 12 months, have you gotten into TROUBLE while you were using alcohol or drugs?	Yes<br>No	dd_trbl
95	CRAFFT Scale	During the past 12 months, have you ever ridden in a CAR driven by someone (including yourself) who was "high" or had been using alcohol or drugs?	Yes<br>No	dd_car
**Recovery and Problem Resolution**				
96	Recovery and Problem Resolution	Besides nicotine, did you used to have a problem with drugs or alcohol, but no longer do?	Yes<br>No	rec_prob
97	Recovery and Problem Resolution	With which substance do you no longer have a problem? (select all that apply) Does not apply	Yes<br>No	rec_na
98	Recovery and Problem Resolution	With which substance do you no longer have a problem? (select all that apply) Alcohol	Yes<br>No	rec_alc

99	Recovery and Problem Resolution	With which substance do you no longer have a problem? (select all that apply) Marijuana	Yes<br>No	rec_pot
100	Recovery and Problem Resolution	With which substance do you no longer have a problem? (select all that apply) Opioids	Yes<br>No	rec_opmed
101	Recovery and Problem Resolution	With which substance do you no longer have a problem? (select all that apply) Other substance	Yes<br>No	rec_othr
102	Recovery and Problem Resolution	Do you consider yourself to be in recovery?	Yes<br>No	rec_self
**Parental Communication and Monitoring**				
103	Parental Communication and Monitoring	My family has clear rules about alcohol and drug use.	Yes<br>No	fna_drul
104	Parental Communication and Monitoring	In the past year, have your parents/guardians talked with you about not drinking and driving or riding with a drunk driver?	Yes<br>No	pg_drnk
105	Parental Communication and Monitoring	If you drank some beer, wine, or liquor (e.g., vodka, whiskey, or gin) without your parents' permission, would you be caught by your parents?	Never<br>Sometimes<br>Most of the time<br>Always	fna_alc
106	Parental Communication and Monitoring	If you go to a party where alcohol is served, would you be caught by your parents?	Never<br>Sometimes<br>Most of the time<br>Always	fna_prty
107	Parental Communication and Monitoring	If you drank and drove, would you be caught by your parents/guardians?	Never<br>Sometimes<br>Most of the time<br>Always	pg_drdr
108	Parental Communication and Monitoring	If you rode in a car driven by a teen driver who had been drinking, would you be caught by your parents/guardians?	Never<br>Sometimes<br>Most of the time<br>Always	pg_drtn
109	Parental Communication and Monitoring	When I am not at home, one of my parents/guardians knows where I am and who I am with.	Never<br>Sometimes<br>Most of the time<br>Always	pg_wher
110	Parental Communication and Monitoring	My parents/guardians ask if I've gotten my homework done.	Never<br>Sometimes<br>Most of the time<br>Always	pg_hwrk
111	Parental Communication and Monitoring	Would your parents/guardians know if you did not come home on time?	Never<br>Sometimes<br>Most of the time<br>Always	pg_time
**Safety**				
112	Safety	How many times in the past year (12 months) have you: been in a physical fight?	Never<br>1-2 times<br>3-5 times<br>6 or more times	pyr_fght

113	Safety	How many times in the past year (12 months) have you: carried a weapon such as a handgun, knife, or club?	Never<br>1-2 times<br>3-5 times<br>6 or more times	pyr_weapt
114	Safety	How many times in the past year (12 months) have you: sold illegal drugs?	Never<br>1-2 times<br>3-5 times<br>6 or more times	pyr_selft
115	Safety	How many times in the past year (12 months) have you: been drunk or high at school?	Never<br>1-2 times<br>3-5 times<br>6 or more times	pyr_dscht
**Bullying**				
116	Bullying	In the past 12 months at school, how often have you been bullied, harassed, or made fun of because of: what someone assumed about your religion, sexual orientation, or race/ethnicity?	Never<br>1-2 times<br>3-5 times<br>6 or more times	bly_bias
117	Bullying	In the past 12 months at school, how often have you been bullied, harassed, or made fun of because of: your appearance or a disability?	Never<br>1-2 times<br>3-5 times<br>6 or more times	bly_dis
118	Bullying	During the past 12 months, has another student at school: bullied you by calling you names?	Yes<br>No	bly_name
119	Bullying	During the past 12 months, has another student at school: threatened to hurt you?	Yes<br>No	bly_thrt
120	Bullying	During the past 12 months, has another student at school: bullied you by hitting, punching, kicking, or pushing you?	Yes<br>No	bly_hit
121	Bullying	During the past 12 months, has another student at school: bullied, harrassed, or spread rumors about you on the Internet, social media, or through text messages?	Yes<br>No	bly_cyb
**Gambling**				
122	Gambling	During the past 12 months, how often have you bet/gambled for money in the following ways? At a gambling machine in a bar, restaurant, gas station, or gambling establishment	Never<br>Less than once a month<br>1-3 times per month<br>Once a week or more	gmb_mach
123	Gambling	During the past 12 months, how often have you bet/gambled for money in the following ways? Online (internet) gambling	Never<br>Less than once a month<br>1-3 times per month<br>Once a week or more	gmb_onl
**Academic Grades**				

124	Academic Grades	Putting them all together, what were your grades like for the last year?	Mostly A<br>Mostly A and B<br>Mostly B<br>Mostly B and C<br>Mostly C<br>Mostly C and D<br>Mostly D<br>Mostly F	grade_yr
**Teacher/Adult Support at School**				
125	Teacher/Adult Support at School	How true are the following statements? At my school, there is a teacher or some other adult: who really cares about me.	Not at all true<br>A little true<br>Pretty much true<br>Very much true	sta_care
126	Teacher/Adult Support at School	How true are the following statements? At my school, there is a teacher or some other adult: who notices when I'm not there.	Not at all true<br>A little true<br>Pretty much true<br>Very much true	sta_notc
127	Teacher/Adult Support at School	How true are the following statements? At my school, there is a teacher or some other adult: who listens to me when I have something to say.	Not at all true<br>A little true<br>Pretty much true<br>Very much true	sta_lstn
128	Teacher/Adult Support at School	How true are the following statements? At my school, there is a teacher or some other adult: who notices if I have trouble learning something.	Not at all true<br>A little true<br>Pretty much true<br>Very much true	sta_tlrn
129	Teacher/Adult Support at School	How true are the following statements? At my school, there is a teacher or some other adult: who tells me when I do a good job.	Not at all true<br>A little true<br>Pretty much true<br>Very much true	sta_gjob
130	Teacher/Adult Support at School	How true are the following statements? At my school, there is a teacher or some other adult: who always wants me to do my best.	Not at all true<br>A little true<br>Pretty much true<br>Very much true	sta_best
131	Teacher/Adult Support at School	How true are the following statements? At my school, there is a teacher or some other adult: who believes I will be a success.	Not at all true<br>A little true<br>Pretty much true<br>Very much true	sta_bels
132	Teacher/Adult Support at School	How true are the following statements? At my school, there is a teacher or some other adult: who encourages me to work hard in school.	Not at all true<br>A little true<br>Pretty much true<br>Very much true	sta_encg
**School Climate**				
133	School Climate	How true are the following statements? At school, I do interesting activities.	Not at all true<br>A little true<br>Pretty much true<br>Very much true	sc_int
134	School Climate	How true are the following statements? At school, I help decide things like class activities or rules.	Not at all true<br>A little true<br>Pretty much true<br>Very much true	sc_dec

135	School Climate	How true are the following statements? At school, I do things that make a difference.	Not at all true<br>A little true<br>Pretty much true<br>Very much true	sc_diff
136	School Climate	How strongly do you agree or disagree with the following statements about your school? I feel close to people at this school.	Strongly disagree<br>Disagree<br>Neither agree nor disagree<br>Agree<br>Strongly agree	sc_close
137	School Climate	How strongly do you agree or disagree with the following statements about your school? I am happy to be at this school.	Strongly disagree<br>Disagree<br>Neither agree nor disagree<br>Agree<br>Strongly agree	sc_happy
138	School Climate	How strongly do you agree or disagree with the following statements about your school? I feel safe in my school.	Strongly disagree<br>Disagree<br>Neither agree nor disagree<br>Agree<br>Strongly agree	sc_safe
139	School Climate	How strongly do you agree or disagree with the following statements about your school? The teachers at this school treat students fairly.	Strongly disagree<br>Disagree<br>Neither agree nor disagree<br>Agree<br>Strongly agree	sc_fair
**Food Insecurity**				
140	Food Insecurity	During the past 30 days, how often did you go hungry because there was not enough food in your home?	Never<br>Rarely<br>Sometimes<br>Most of the time<br>Always	hunger
